# Dibromido(6,6′-dimethyl-2,2′-bipyridine-κ^2^ *N*,*N*′)cadmium

**DOI:** 10.1107/S1600536812033648

**Published:** 2012-08-04

**Authors:** Sadif A. Shirvan, Sara Haydari Dezfuli

**Affiliations:** a Department of Chemistry, Islamic Azad University, Omidieh Branch, Omidieh, Iran

## Abstract

In the title compound, [CdBr_2_(C_12_H_12_N_2_)], the Cd^II^ atom is four-coordinated in a distorted tetra­hedral geometry by two N atoms from a 6,6′-dimethyl-2,2′-bipyridine ligand and two terminal Br atoms. In the crystal, C—H⋯Br hydrogen bonds and π–π stacking inter­actions between the pyridine rings [centroid–centroid distance = 3.763 (5) Å] are present.

## Related literature

For related structures, see: Akbarzadeh Torbati *et al.* (2010 ▶); Alizadeh *et al.* (2010 ▶, 2011 ▶); Alizadeh, Kalateh, Ebadi *et al.* (2009 ▶); Alizadeh, Kalateh, Khoshtarkib *et al.* (2009 ▶); Alizadeh, Khoshtarkib *et al.* (2009 ▶); Itoh *et al.* (2005 ▶); Kou *et al.* (2008 ▶); Onggo *et al.* (2005 ▶); Shirvan & Haydari Dezfuli (2012*a* ▶,*b* ▶).
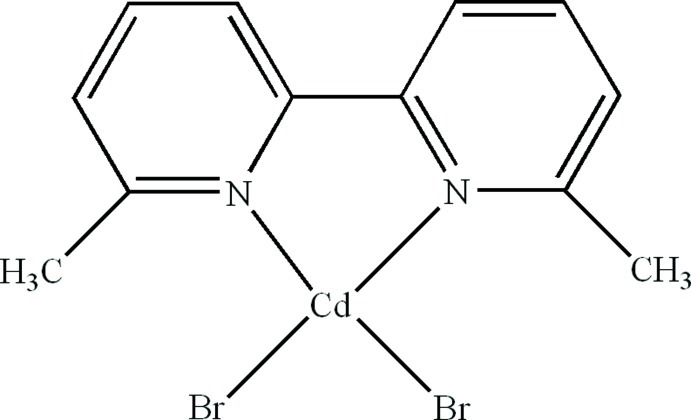


## Experimental

### 

#### Crystal data

[CdBr_2_(C_12_H_12_N_2_)]
*M* *_r_* = 456.45
Monoclinic, 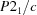

*a* = 7.7606 (11) Å
*b* = 10.3832 (17) Å
*c* = 18.184 (2) Å
β = 97.460 (11)°
*V* = 1452.8 (4) Å^3^
*Z* = 4
Mo *K*α radiation
μ = 6.98 mm^−1^
*T* = 298 K
0.40 × 0.20 × 0.15 mm

#### Data collection

Bruker APEXII CCD diffractometer
Absorption correction: multi-scan (*SADABS*; Bruker, 2001 ▶) *T*_min_ = 0.054, *T*_max_ = 0.155
11844 measured reflections
2862 independent reflections
1945 reflections with *I* > 2σ(*I*)
*R*_int_ = 0.098

#### Refinement

*R*[*F* ^2^ > 2σ(*F* ^2^)] = 0.047
*wR*(*F* ^2^) = 0.124
*S* = 1.03
2862 reflections
154 parameters
H-atom parameters constrained
Δρ_max_ = 0.61 e Å^−3^
Δρ_min_ = −0.78 e Å^−3^

### 

Data collection: *APEX2* (Bruker, 2007 ▶); cell refinement: *SAINT* (Bruker, 2007 ▶); data reduction: *SAINT*; program(s) used to solve structure: *SHELXS97* (Sheldrick, 2008 ▶); program(s) used to refine structure: *SHELXL97* (Sheldrick, 2008 ▶); molecular graphics: *ORTEP-3* (Farrugia, 1997 ▶) and *Mercury* (Macrae *et al.*, 2006 ▶); software used to prepare material for publication: *SHELXTL* (Sheldrick, 2008 ▶).

## Supplementary Material

Crystal structure: contains datablock(s) I, global. DOI: 10.1107/S1600536812033648/hy2576sup1.cif

Structure factors: contains datablock(s) I. DOI: 10.1107/S1600536812033648/hy2576Isup2.hkl

Additional supplementary materials:  crystallographic information; 3D view; checkCIF report

## Figures and Tables

**Table 1:** Hydrogen-bond geometry (Å, °)

*D*—H⋯*A*	*D*—H	H⋯*A*	*D*⋯*A*	*D*—H⋯*A*
C1—H1*C*⋯Br1^i^	0.96	2.90	3.848 (10)	171

Symmetry code: (i) 
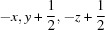
.

**Fig. 1.**
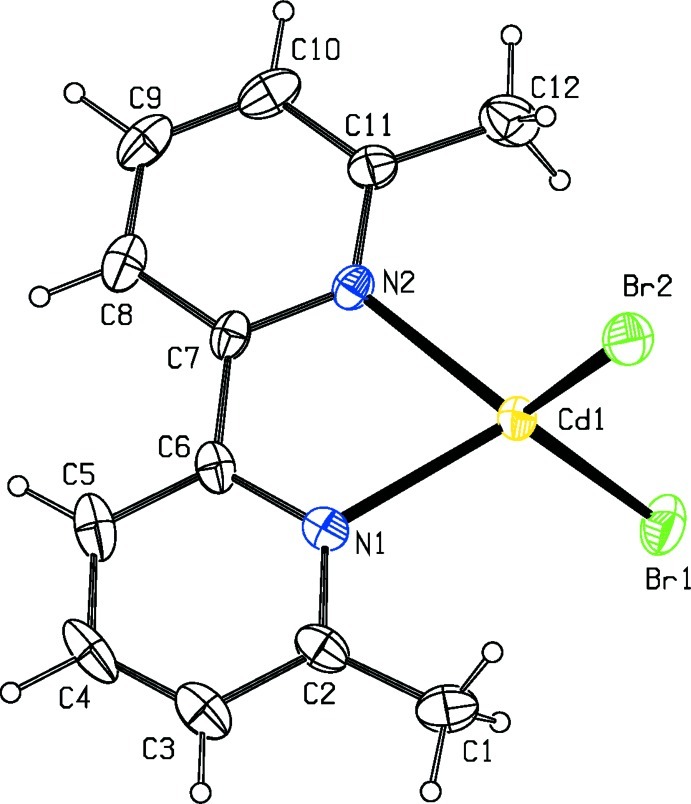
The molecular structure of the title compound. Displacement ellipsoids are drawn at the 30% probability level.

**Fig. 2.**
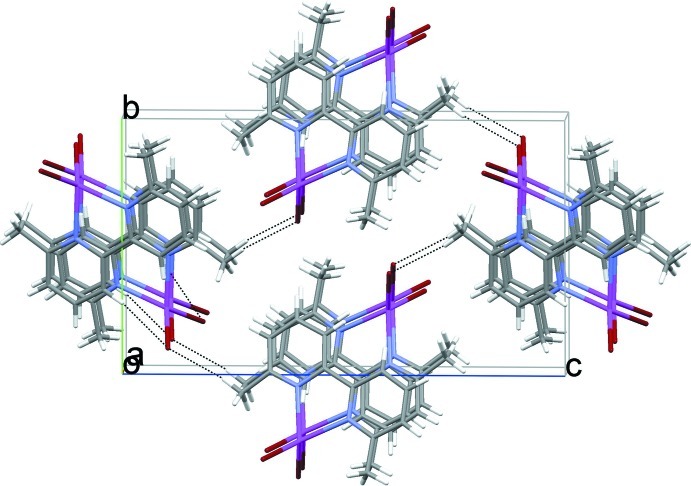
Crystal packing diagram of the title compound. Hydrogen bonds are shown as dashed lines.

## supplementary crystallographic information

### Comment

Recently, we reported the synthesis and crystal structures of [Cd(5,5'-dmbpy)(µ-Br)_2_]_n_ (Shirvan & Haydari Dezfuli, 2012*a*) and [CdBr_2_(4,4'-dmbpy)(DMSO)] (Shirvan & Haydari Dezfuli, 2012*b*) (5,5'-dmbpy = 5,5'-dimethyl-2,2'-bipyridine, 4,4'-dmbpy = 4,4'-dimethyl-2,2'-bipyridine, DMSO = dimethyl sulfoxide). 6,6'-Dimethyl-2,2'-bipyridine (6,6'-dmbipy) is a good bidentate ligand and numerous complexes with 6,6'-dmbipy have been prepared, such as that of zinc (Alizadeh, Kalateh, Ebadi *et al.*, 2009; Alizadeh, Kalateh, Khoshtarkib *et al.*, 2009; Alizadeh, Khoshtarkib *et al.*, 2009), copper (Itoh *et al.*, 2005), cadmium (Alizadeh *et al.*, 2010), cobalt (Akbarzadeh Torbati *et al.*, 2010), nickel (Kou *et al.*, 2008), ruthenium (Onggo *et al.*, 2005) and mercury (Alizadeh *et al.*, 2011). We report herein the synthesis and crystal structure of the title compound.

In the title compound (Fig. 1), the Cd^II^ atom is four-coordinated in a distorted tetrahedral geometry by two N atoms from a 6,6'-dimethyl-2,2'-bipyridine ligand and two terminal Br atoms. The crystal structure is stabilized by intermolecular C—H···Br hydrogen bonds (Table 1) and π–π contacts (Fig. 2) between the pyridine rings, *Cg*2···*Cg*3^i^ [symmetry code: (i) -x, 1-y, -z. *Cg*2 and *Cg*3 are the centroids of the N1/C2–C6 ring and N2/C7–C11 ring], with a centroid–centroid distance of 3.763 (5) Å.

### Experimental

For the preparation of the title compound, a solution of 6,6'-dimethyl-2,2'-bipyridine (0.25 g, 1.33 mmol) in methanol (10 ml) was added to a solution of CdBr_2_.4H_2_O (0.46 g, 1.33 mmol) in methanol (10 ml) at room temperature. Crystals suitable for X-ray diffraction experiment were obtained by methanol diffusion into a colorless solution in DMSO after one week (yield: 0.47 g, 77.4%).

### Refinement

H atoms were positioned geometrically and refined as riding atoms, with C—H = 0.93 (aromatic) and 0.96 (CH_3_) Å and with *U*_iso_(H) = 1.2*U*_eq_(C).

### Crystal data

**Table:**

[CdBr_2_(C_12_H_12_N_2_)]	*F*(000) = 864
*M**_r_* = 456.45	*D*_x_ = 2.087 Mg m^−^^3^
Monoclinic, *P*2_1_/*c*	Mo *K*α radiation, λ = 0.71073 Å
Hall symbol: -P 2ybc	Cell parameters from 11844 reflections
*a* = 7.7606 (11) Å	θ = 2.3–26.0°
*b* = 10.3832 (17) Å	µ = 6.98 mm^−^^1^
*c* = 18.184 (2) Å	*T* = 298 K
β = 97.460 (11)°	Block, colorless
*V* = 1452.8 (4) Å^3^	0.40 × 0.20 × 0.15 mm
*Z* = 4	

### Data collection

**Table:**

Bruker APEXII CCD diffractometer	2862 independent reflections
Radiation source: fine-focus sealed tube	1945 reflections with *I* > 2σ(*I*)
Graphite monochromator	*R*_int_ = 0.098
φ and ω scans	θ_max_ = 26.0°, θ_min_ = 2.3°
Absorption correction: multi-scan (*SADABS*; Bruker, 2001)	*h* = −9→9
*T*_min_ = 0.054, *T*_max_ = 0.155	*k* = −12→12
11844 measured reflections	*l* = −19→22

### Refinement

**Table:**

Refinement on *F*^2^	Primary atom site location: structure-invariant direct methods
Least-squares matrix: full	Secondary atom site location: difference Fourier map
*R*[*F*^2^ > 2σ(*F*^2^)] = 0.047	Hydrogen site location: inferred from neighbouring sites
*wR*(*F*^2^) = 0.124	H-atom parameters constrained
*S* = 1.03	*w* = 1/[σ^2^(*F*_o_^2^) + (0.0602*P*)^2^] where *P* = (*F*_o_^2^ + 2*F*_c_^2^)/3
2862 reflections	(Δ/σ)_max_ = 0.003
154 parameters	Δρ_max_ = 0.61 e Å^−^^3^
0 restraints	Δρ_min_ = −0.78 e Å^−^^3^

### Special details

**Table:**

Geometry. All e.s.d.'s (except the e.s.d. in the dihedral angle between two l.s. planes) are estimated using the full covariance matrix. The cell e.s.d.'s are taken into account individually in the estimation of e.s.d.'s in distances, angles and torsion angles; correlations between e.s.d.'s in cell parameters are only used when they are defined by crystal symmetry. An approximate (isotropic) treatment of cell e.s.d.'s is used for estimating e.s.d.'s involving l.s. planes.
Refinement. Refinement of *F*^2^ against ALL reflections. The weighted *R*-factor *wR* and goodness of fit *S* are based on *F*^2^, conventional *R*-factors *R* are based on *F*, with *F* set to zero for negative *F*^2^. The threshold expression of *F*^2^ > σ(*F*^2^) is used only for calculating *R*-factors(gt) *etc*. and is not relevant to the choice of reflections for refinement. *R*-factors based on *F*^2^ are statistically about twice as large as those based on *F*, and *R*- factors based on ALL data will be even larger.

### Fractional atomic coordinates and isotropic or equivalent isotropic displacement parameters (Å^2^)

**Table:**

	*x*	*y*	*z*	*U*_iso_*/*U*_eq_	
C1	0.1386 (14)	0.4345 (10)	0.2292 (5)	0.081 (3)	
H1A	0.2494	0.3951	0.2444	0.098*	
H1B	0.0530	0.3689	0.2161	0.098*	
H1C	0.1055	0.4852	0.2693	0.098*	
C2	0.1505 (10)	0.5178 (8)	0.1648 (5)	0.060 (2)	
C3	0.1116 (12)	0.6486 (9)	0.1659 (6)	0.077 (3)	
H3	0.0782	0.6861	0.2083	0.092*	
C4	0.1230 (12)	0.7210 (8)	0.1044 (7)	0.078 (3)	
H4	0.0963	0.8083	0.1044	0.094*	
C5	0.1750 (11)	0.6642 (8)	0.0409 (6)	0.071 (3)	
H5	0.1835	0.7133	−0.0013	0.085*	
C6	0.2136 (9)	0.5334 (7)	0.0417 (4)	0.0483 (17)	
C7	0.2699 (8)	0.4638 (7)	−0.0232 (4)	0.0461 (17)	
C8	0.2878 (11)	0.5254 (9)	−0.0896 (5)	0.066 (2)	
H8	0.2613	0.6124	−0.0958	0.079*	
C9	0.3445 (11)	0.4568 (11)	−0.1454 (5)	0.072 (3)	
H9	0.3569	0.4969	−0.1902	0.087*	
C10	0.3835 (11)	0.3288 (11)	−0.1359 (5)	0.070 (3)	
H10	0.4226	0.2818	−0.1740	0.084*	
C11	0.3646 (10)	0.2703 (8)	−0.0699 (5)	0.0543 (19)	
C12	0.4037 (14)	0.1324 (10)	−0.0560 (6)	0.080 (3)	
H12A	0.3013	0.0891	−0.0446	0.095*	
H12B	0.4944	0.1241	−0.0150	0.095*	
H12C	0.4410	0.0945	−0.0995	0.095*	
N1	0.1993 (7)	0.4635 (6)	0.1027 (3)	0.0470 (14)	
N2	0.3092 (7)	0.3379 (6)	−0.0136 (3)	0.0448 (13)	
Cd1	0.27194 (7)	0.25065 (5)	0.09801 (3)	0.04813 (18)	
Br1	0.01860 (13)	0.10203 (9)	0.10288 (6)	0.0775 (3)	
Br2	0.54796 (12)	0.18801 (10)	0.17765 (5)	0.0693 (3)	

### Atomic displacement parameters (Å^2^)

**Table:**

	*U*^11^	*U*^22^	*U*^33^	*U*^12^	*U*^13^	*U*^23^
C1	0.105 (7)	0.093 (7)	0.049 (5)	0.005 (6)	0.024 (5)	−0.017 (5)
C2	0.053 (5)	0.056 (5)	0.069 (6)	0.005 (4)	−0.001 (4)	−0.020 (4)
C3	0.074 (6)	0.058 (6)	0.095 (8)	0.011 (5)	−0.002 (5)	−0.019 (5)
C4	0.064 (5)	0.040 (5)	0.123 (9)	0.016 (4)	−0.022 (6)	−0.027 (5)
C5	0.063 (5)	0.042 (4)	0.099 (7)	−0.005 (4)	−0.023 (5)	0.011 (5)
C6	0.042 (4)	0.039 (4)	0.060 (5)	−0.002 (3)	−0.010 (3)	0.004 (3)
C7	0.039 (4)	0.049 (4)	0.048 (4)	−0.011 (3)	−0.003 (3)	0.011 (3)
C8	0.062 (5)	0.069 (6)	0.066 (6)	−0.011 (4)	0.002 (4)	0.023 (5)
C9	0.066 (5)	0.103 (8)	0.048 (5)	−0.017 (5)	0.006 (4)	0.020 (5)
C10	0.060 (5)	0.109 (8)	0.042 (4)	−0.019 (5)	0.007 (4)	−0.003 (5)
C11	0.055 (4)	0.060 (5)	0.048 (4)	−0.002 (4)	0.009 (4)	−0.005 (4)
C12	0.093 (7)	0.079 (7)	0.067 (6)	0.007 (5)	0.013 (5)	−0.019 (5)
N1	0.042 (3)	0.048 (3)	0.051 (4)	−0.001 (3)	0.002 (3)	−0.007 (3)
N2	0.047 (3)	0.046 (3)	0.041 (3)	−0.008 (3)	0.004 (3)	0.001 (3)
Cd1	0.0576 (3)	0.0421 (3)	0.0456 (3)	0.0049 (3)	0.0102 (2)	0.0069 (2)
Br1	0.0804 (6)	0.0631 (6)	0.0900 (7)	−0.0163 (5)	0.0155 (5)	0.0197 (5)
Br2	0.0661 (5)	0.0808 (6)	0.0597 (5)	0.0161 (5)	0.0033 (4)	0.0171 (5)

### Geometric parameters (Å, º)

**Table:**

C1—C2	1.469 (13)	C8—C9	1.358 (14)
C1—H1A	0.9600	C8—H8	0.9300
C1—H1B	0.9600	C9—C10	1.369 (15)
C1—H1C	0.9600	C9—H9	0.9300
C2—N1	1.360 (10)	C10—C11	1.369 (12)
C2—C3	1.392 (13)	C10—H10	0.9300
C3—C4	1.360 (15)	C11—N2	1.357 (10)
C3—H3	0.9300	C11—C12	1.479 (12)
C4—C5	1.402 (14)	C12—H12A	0.9600
C4—H4	0.9300	C12—H12B	0.9600
C5—C6	1.390 (11)	C12—H12C	0.9600
C5—H5	0.9300	N1—Cd1	2.285 (6)
C6—N1	1.341 (10)	N2—Cd1	2.274 (6)
C6—C7	1.498 (11)	Cd1—Br1	2.5099 (11)
C7—N2	1.349 (9)	Cd1—Br2	2.5106 (11)
C7—C8	1.389 (11)		
			
C2—C1—H1A	109.5	C8—C9—C10	120.1 (8)
C2—C1—H1B	109.5	C8—C9—H9	119.9
H1A—C1—H1B	109.5	C10—C9—H9	119.9
C2—C1—H1C	109.5	C9—C10—C11	119.6 (9)
H1A—C1—H1C	109.5	C9—C10—H10	120.2
H1B—C1—H1C	109.5	C11—C10—H10	120.2
N1—C2—C3	120.1 (9)	N2—C11—C10	120.8 (8)
N1—C2—C1	118.2 (7)	N2—C11—C12	116.8 (7)
C3—C2—C1	121.8 (9)	C10—C11—C12	122.4 (8)
C4—C3—C2	119.3 (10)	C11—C12—H12A	109.5
C4—C3—H3	120.3	C11—C12—H12B	109.5
C2—C3—H3	120.3	H12A—C12—H12B	109.5
C3—C4—C5	120.1 (8)	C11—C12—H12C	109.5
C3—C4—H4	119.9	H12A—C12—H12C	109.5
C5—C4—H4	119.9	H12B—C12—H12C	109.5
C6—C5—C4	119.1 (9)	C6—N1—C2	121.6 (7)
C6—C5—H5	120.5	C6—N1—Cd1	116.5 (5)
C4—C5—H5	120.5	C2—N1—Cd1	121.9 (5)
N1—C6—C5	119.8 (8)	C7—N2—C11	119.4 (6)
N1—C6—C7	117.0 (6)	C7—N2—Cd1	116.7 (5)
C5—C6—C7	123.1 (8)	C11—N2—Cd1	123.8 (5)
N2—C7—C8	120.9 (7)	N2—Cd1—N1	73.0 (2)
N2—C7—C6	116.7 (6)	N2—Cd1—Br1	117.85 (15)
C8—C7—C6	122.4 (7)	N1—Cd1—Br1	113.28 (14)
C9—C8—C7	119.1 (9)	N2—Cd1—Br2	114.84 (15)
C9—C8—H8	120.4	N1—Cd1—Br2	115.17 (15)
C7—C8—H8	120.4	Br1—Cd1—Br2	115.72 (4)
			
N1—C2—C3—C4	0.0 (13)	C3—C2—N1—Cd1	178.3 (6)
C1—C2—C3—C4	−179.3 (9)	C1—C2—N1—Cd1	−2.4 (10)
C2—C3—C4—C5	−0.6 (15)	C8—C7—N2—C11	−0.9 (10)
C3—C4—C5—C6	0.3 (14)	C6—C7—N2—C11	−178.6 (7)
C4—C5—C6—N1	0.6 (12)	C8—C7—N2—Cd1	−179.7 (5)
C4—C5—C6—C7	179.9 (7)	C6—C7—N2—Cd1	2.6 (7)
N1—C6—C7—N2	−3.0 (9)	C10—C11—N2—C7	0.9 (11)
C5—C6—C7—N2	177.7 (7)	C12—C11—N2—C7	−179.4 (7)
N1—C6—C7—C8	179.3 (7)	C10—C11—N2—Cd1	179.7 (6)
C5—C6—C7—C8	0.0 (11)	C12—C11—N2—Cd1	−0.6 (10)
N2—C7—C8—C9	0.4 (12)	C7—N2—Cd1—N1	−1.2 (4)
C6—C7—C8—C9	178.0 (7)	C11—N2—Cd1—N1	180.0 (6)
C7—C8—C9—C10	−0.1 (13)	C7—N2—Cd1—Br1	106.6 (4)
C8—C9—C10—C11	0.2 (13)	C11—N2—Cd1—Br1	−72.2 (6)
C9—C10—C11—N2	−0.6 (13)	C7—N2—Cd1—Br2	−111.6 (4)
C9—C10—C11—C12	179.8 (8)	C11—N2—Cd1—Br2	69.6 (6)
C5—C6—N1—C2	−1.2 (11)	C6—N1—Cd1—N2	−0.5 (5)
C7—C6—N1—C2	179.4 (6)	C2—N1—Cd1—N2	−178.0 (6)
C5—C6—N1—Cd1	−178.8 (5)	C6—N1—Cd1—Br1	−114.1 (5)
C7—C6—N1—Cd1	1.9 (8)	C2—N1—Cd1—Br1	68.4 (6)
C3—C2—N1—C6	0.9 (11)	C6—N1—Cd1—Br2	109.5 (5)
C1—C2—N1—C6	−179.8 (8)	C2—N1—Cd1—Br2	−68.0 (5)

### Hydrogen-bond geometry (Å, º)

**Table:**

*D*—H···*A*	*D*—H	H···*A*	*D*···*A*	*D*—H···*A*
C1—H1*C*···Br1^i^	0.96	2.90	3.848 (10)	171

Symmetry code: (i) −*x*, *y*+1/2, −*z*+1/2.
